# Development of a Point-of-Care System Based on White Light Reflectance Spectroscopy: Application in CRP Determination

**DOI:** 10.3390/bios11080268

**Published:** 2021-08-08

**Authors:** Dimitra Tsounidi, Georgios Koukouvinos, Vasilios Christianidis, Evangelia Legaki, Vasiliki Giogli, Konstantina Panagiotopoulou, Styliani Taka, Zoi Ekaterinidi, Sotirios Kakabakos, Ioannis Raptis, Panagiota Petrou

**Affiliations:** 1 Immunoassay/Immunosensors Lab, Institute of Nuclear & Radiological Sciences & Technology, Energy & Safety, National Centre for Scientific Research ‘‘Demokritos”, 15310 Aghia Paraskevi, Greece; dtsounidi@rrp.demokritos.gr (D.T.); geokoukoubinos@yahoo.gr (G.K.); skakab@rrp.demokritos.gr (S.K.); 2 ThetaMetrisis S.A., Polydefkous 14, 12243 Egaleo, Greece; v.christianidis@thetametrisis.com (V.C.); i.raptis@inn.demokritos.gr (I.R.); 3 STARTBIO Molecular Diagnostics and Biotechnology Services P.C., 11527 Athens, Greece; evangelialegaki@gmail.com (E.L.); vickigiogli@gmail.com (V.G.); kon_na_panagiotopoulou@hotmail.com (K.P.); takastella@hotmail.com (S.T.); 4 Software Competitiveness International GmbH, 64293 Darmstadt, Germany; zoi.ekaterinidi@softcom-int.com; 5 Institute of Nanoscience and Nanotechnology, National Centre for Scientific Research ‘‘Demokritos”, 15310 Aghia Paraskevi, Greece

**Keywords:** C-Reactive protein, point-of care, white light reflectance spectroscopy, label-free detection

## Abstract

The development of methods and miniaturized systems for fast and reliable quantitative determinations at the Point-of-Care is a top challenge and priority in diagnostics. In this work, a compact bench-top system, based on White Light Reflectance Spectroscopy, is introduced and evaluated in an application with high clinical interest, namely the determination of C-Reactive protein (CRP) in human blood samples. The system encompassed all the necessary electronic and optical components for the performance of the assay, while the dedicated software provided the sequence and duration of assay steps, the reagents flow rate, the real-time monitoring of sensor response, and data processing to deliver in short time and accurately the CPR concentration in the sample. The CRP assay included two steps, the first comprising the binding of sample CRP onto the chip immobilized capture antibody and the second the reaction of the surface immunosorbed CRP molecules with the detection antibody. The assay duration was 12 min and the dynamic range was from 0.05 to 200 μg/mL, covering both normal values and acute inflammation incidents. There was an excellent agreement between CRP values determined in human plasma samples using the developed device with those received for the same samples by a standard diagnostic laboratory method.

## 1. Introduction

C-Reactive Protein (CRP), a 118 kDa molecular weight protein, is the most established biomarker of inflammation. It consists of five non-covalently bound identical subunits and belongs to the class of acute phase proteins [1]. The production of CRP is carried out mainly by hepatocytes and it is strongly regulated by the pro-inflammatory cytokines, especially interleukin-6 [1,2]. While in healthy individuals CRP levels are below 5 μg/mL, they can increase rapidly up to 1000-fold within 24–48 h after the onset of an acute inflammatory condition. Moreover, rapid decrease in CRP levels within the first 48 h of therapy has been correlated with an effective response to the treatment, while persistently high concentrations have been related to high mortality rates [3,4]. In addition, CRP can be used to distinguish bacterial from viral infection since its levels are much higher in case of bacterial infections, rather than in viral ones. It is also widely used as a biomarker for sepsis diagnosis, which is one of the major mortality causes in critically ill patients [4,5]. Elevated CRP levels are related to the severity of sepsis and the prognosis of the disease outcome [6,7]. Moreover, it is the most widely used biomarker for the early detection of sepsis in neonates. In this case, CRP serum concentration rises above 5 μg/mL in about 6 h after stimulation and peaks 48 h after sepsis onset, while studies have demonstrated the importance of repetitive CRP measurements in monitoring the treatment response in infected neonates [3,8]. Nonetheless, the role of CRP as a biomarker of inflammation extends beyond microbial infections. CRP levels are also significantly elevated in case of trauma, injury and tissue necrosis, diabetes, cancer, and rheumatic diseases [2,4,9]. Moreover, mildly elevated CRP levels at 1–3 μg/mL have been associated with high risk for cardiovascular diseases and acute myocardial infraction [2,10]. Therefore, the quantitative and accurate determination of CRP concentration in blood samples is one of the most commonly performed test in clinical settings to confirm an inflammatory condition and monitor the patient response to therapy.

Nowadays, CRP determination is performed by a variety of immunochemical methods [11,12], such as immunoturbidimetric assays [13], enzyme-linked immunosorbent assays [14], chemiluminescence [15] and fluorescence immunoassays [16]. These methods provide high sensitivity and accuracy; however, because they require special equipment and skilled personnel, they are performed mainly at hospitals’ central laboratories, which might lead to increased workflow and delays in the delivery of tests results [17,18]. Consequently, the current challenge in diagnostics field is to overcome the limitations of conventional techniques by the development of methods and devices that can provide fast and reliable quantitative results at the Point-of-Care (PoC). In this way, early diagnosis can be achieved through swift medical decisions and timely treatment initiation [19,20,21]. To this direction, different types of biosensors [12,18,22,23,24], based mainly on electrochemical [25,26,27] or optical transducers [28,29,30], have been proposed for CRP determination. Furthermore, portable and bench-top analyzers are commercially available and provide very fast CRP detection [12,17,31].

In a previous report, an optical label-free immunosensor based on White Light Reflectance Spectroscopy (WLRS) has been demonstrated for the determination of CRP in whole blood samples [32]. This was achieved through a fast immunoassay using SiO_2_/Si chips coated with a capture antibody and employing a biotinylated detection antibody and streptavidin. The WLRS apparatus used at that study consisted of a small size system accommodating a light source, a miniaturized USB controlled spectrometer and a reflection probe. Nonetheless, an external peristaltic micropump was employed for reagents delivery through manual handling of the assay protocol.

**Figure 1:**
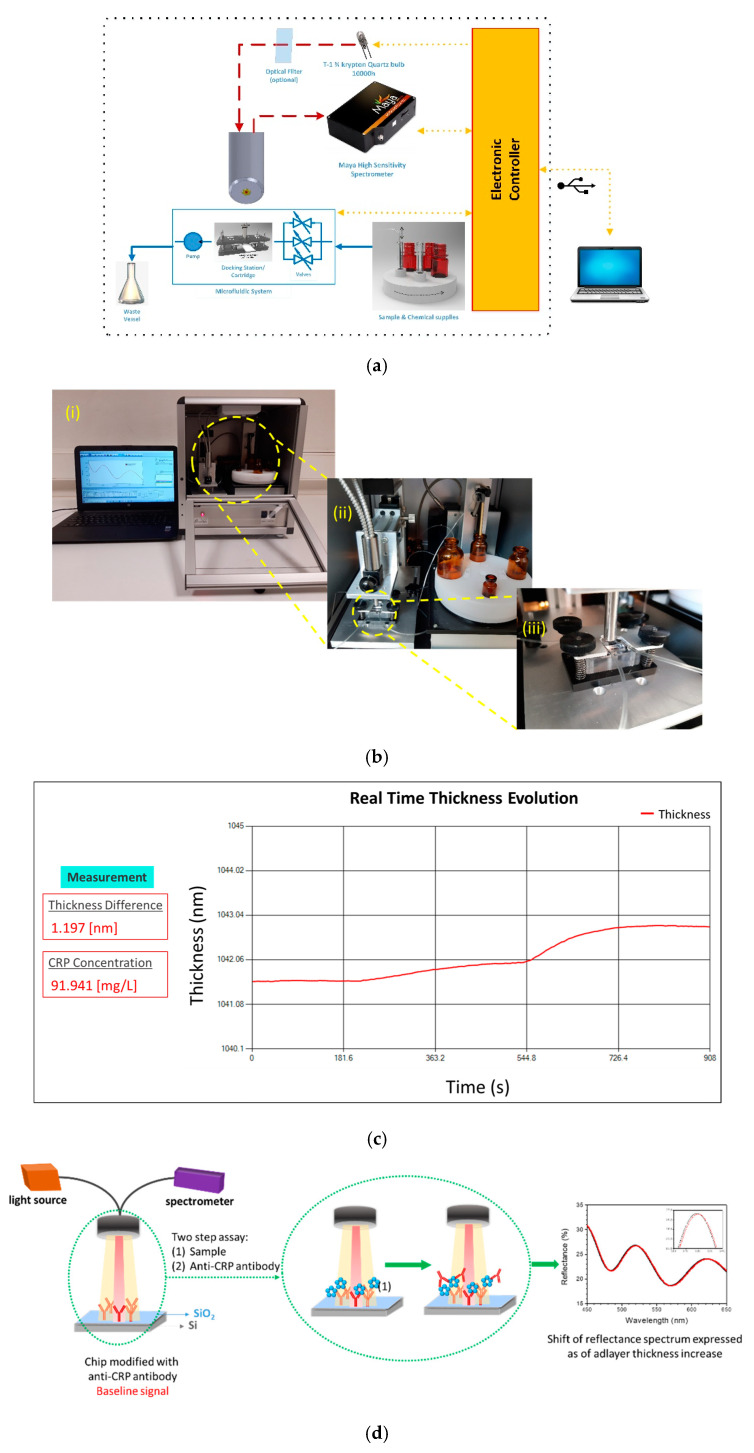
(**a**) Depiction of the main part of the instrument. (**b**) Images of the: (i) whole instrument and accompanying PC, (ii) fluidic module, and (iii) docking station. (**c**) Depiction of the PC screen at the end of a run. (**d**) Schematic of the chip, sensing principle and two-site sandwich immunoassay procedure.

In this work, a compact bench-top bioanalytical system, based on WLRS technology, for the fast and accurate determination of CRP in human serum samples at the Point-of-Care is presented. The hardware part of the system encompasses all the necessary electronic and optical components for the performance of the assay (Figure 1a,b). In particular, the system includes a custom designed docking station that allows the accurate alignment of the biochip with the reflection probe and a flow system employing a peristaltic micropump and a sampler with a rotating base for samples and reagents placing, both controlled by the accompanying software. The dedicated software defines the sequence and duration of the assay steps and the flow rate of reagents, and at the same time provides for the real-time monitoring of the immunosensor response as well as to the mathematical processing of the raw reflectance data to provide the CPR concentration in the sample upon completion of the assay (Figure 1c). The CRP assay is completed in two steps, the first step comprising the binding of sample CRP onto the immobilized onto the chip capture antibody, and the second the reaction of the immunosorbed onto the surface CRP molecules with the detection antibody (Figure 1d). The duration of each step was optimized to reduce as possible the assay duration without affecting the assay analytical performance. Human plasma samples were analyzed with the developed system and the results were compared with those received for the same samples by a standard diagnostic laboratory method. Finally, a comparison of the system developed with commercially available bench-top and handheld instruments is provided that reveals the advantages of the developed system and its potential for wider use at the Point-of-Care.

## 2. Materials and Methods

### 2.1. Materials

Affinity purified goat antibody against human C-Reactive Protein (code GC019) and C-Reactive Protein (CRP) from human fluids were purchased from Scripps Laboratories (San Diego, CA, USA). Monoclonal antibody against CRP (code 6404) was from Medix Biochemica (Espoo, Finland). Goat anti-human CRP serum and goat anti-human CRP IgG fraction were purchased from Sanovo Biotech A/S (Odense, Denmark). Bovine serum albumin (BSA) and (3-aminopropyl)triethoxysilane (APTES) were purchased from Acros Organics (Geel, Belgium). All other reagents were from Merck (Darmstadt, Germany). The water used throughout the study was distilled. Blood samples were obtained in two different vacutainers, one with clot activator for CPR measurement in serum and one with heparin for CPR measurement in plasma. Written informed consent was obtained from donors at the premises of STARTBIO Molecular Diagnostics and Biotechnology Services P.C. (Athens, Greece). All samples were analyzed with the highly sensitive CRP Abbott assay. WLRS chips were fabricated using four-inch device-quality Si wafers purchased from Si-Mat Germany (Kaufering, Germany). The wafers were cleaned by sonication in acetone and isopropanol prior to thermal oxidization at 1100 °C for approximately 4 h at the Nanotechnology and MEMS lab of the Institute of Nanoscience and Nanotechnology of NCSR “Demokritos” to develop a 1000-nm thick SiO_2_ layer. After that, the wafers were diced to chips with dimensions of 5 mm × 15 mm to fit in the docking station of the bioanalytical system.

### 2.2. Instrumentation

The system was comprised of two main parts: the hardware where the bioreactions were taking place and the software that controls the hardware, processes the sensor’s response (reflectance spectrum), and finally provides the CRP concentration in the sample under analysis.

The hardware part of the system consisted of the: optical module, docking station, reagents handling module, and electronic module. The optical module included: (a) a light source emitting in the visible/near-infrared spectral range, (b) a miniaturized spectrometer, and (c) an on-purpose designed reflection probe. The light source was equipped with an electronic circuit for stabilized light emission both in terms of spectral content and intensity to facilitate very long operation (>10,000 h) at clinical settings. The miniaturized spectrometer (Maya 2000Pro, 16-bit A/D) was tuned to operate in the 450–650 nm spectral range. The reflection probe accommodated both the illumination fibers with 200-μm core diameter each and the collecting fiber of the same diameter, and was designed to provide easy installation in the system and fixed secure distance from the biochip. The docking station module allowed for facile insertion of the biochip and its accurate positioning with respect to the reflection probe for the monitoring of the bioreactions (Figure 1b, part III). The docking station was designed to accommodate WLRS biochips with 5 mm × 15 mm size encapsulated with the microfluidic chamber for the supply of the immunoassay reagents. The reagents handling module was responsible for the automated supply of reagent solutions required for immunoassay performance (Figure 1b, part II). It comprised of a carousel unit that could accommodate up to four vials loaded with the solutions of reagents required, a programmable micro-pump for the supply of the solutions at the desired flow-rate, and a z-axis moving sampling probe to draw the reagent from the selected vial. The reagents handling module was controlled by a microcontroller appropriately programmed to allow the user to define the sequence of reagents flow, the duration of each reagent’s flow, as well as the flow rate. The system was accompanied by an electronics module that controls the reagents handling module, the spectrometer, the light source and supplied the communication with the PC through a standard USB port.

The software application consisted of a user-friendly interface to control the PoC system with a simple wizard-based start/stop operation to assist the user to correctly complete the steps required for the assay as well as the collection, the visualization, and the storage of the data. During the assay, the application recorded the reflectance spectrum from the biochip (1 spectrum per s), performed the normalization with respect to dark and reference spectra, and fitted the resulting normalized reflectance spectrum by the appropriate physical model and algorithm for the determination in real time of the mean thickness of the biomolecular layer in the probed area, and visualized the resulting biomolecular adlayer thickness increase. At the end of the assay, the collected data were stored automatically in a file, while the application calculated the CRP concentration in the sample based onto the linear regression equation of the stored calibration curve. For the implementation of the software application the programming language C#.net, windows forms, and the operating system Windows 10 were used. The desktop application integrates the control: a) of the reagents handling module through a script language integrated in a window process, and b) of the optical module through a dynamic link library (dll) wrapper created in C++ which integrated the processing algorithms with the C# application.

### 2.3. Chip Biofunctionalization and Assay Protocol

The WLRS chips were firstly cleaned through successive sonication for 10 min in bath of acetone and 2-propanol, and after drying under a nitrogen flow, they were immersed in a 1:1 H_2_SO_4_/H_2_O_2_ (30% *v*/*v*) mixture for 20 min. After that, they were washed with distilled water and immersed in a 2% *v*/*v* aqueous APTES solution for 20 min, followed by gentle washing with distilled water and drying under a nitrogen flow. Finally, the chips were cured at 120 °C for 20 min, and kept at room temperature (RT) in a desiccator until use. For the biofunctionalization, a 3 × 5 mm^2^ area at the center of the APTES-modified chips was spotted with a 100 μg/mL anti-CRP antibody solution in 0.05 M carbonate buffer, pH 9.2, using the BioOdyssey Calligrapher MiniArrayer (Bio-Rad Laboratories Inc., Hercules, CA, USA). After spotting, the chips were incubated overnight at RT under controlled humidity conditions (75%) and then, they were immersed in blocking solution (1% *w*/*v* BSA in 0.1 M NaHCO_3_, pH 8.5) for 2 h at RT. Finally, the biochips were washed with washing solution (0.01 M Tris-HCl, pH 8.5, 0.9 *w*/*v* NaCl) and dried under a nitrogen flow. The antibody coated and blocked chips, referred to thereafter as biochips, were kept at 4 °C in a desiccator until use. Prior to the assay, the biochips were assembled with the microfluidic cell, placed in the docking station of the device, and the fluidic connections with the reagents handling module were made. The protocol sequence was then initiated by the software. At first, the biochip was equilibrated with assay buffer (0.05 M Tris-HCl, pH 7.8, 0.9% *w*/*v* NaCl, 0.5% *w*/*v* BSA). Then, the CRP calibrators prepared in assay buffer or plasma samples 20-fold diluted with assay buffer were run for 7 min, followed by a 5 μg/mL anti-CRP antibody solution in assay buffer for 5 min. All solutions run at a constant flow rate of 30 μL/min.

## 3. Results

### 3.1. Assay Development and Optimization

**Figure 2:**
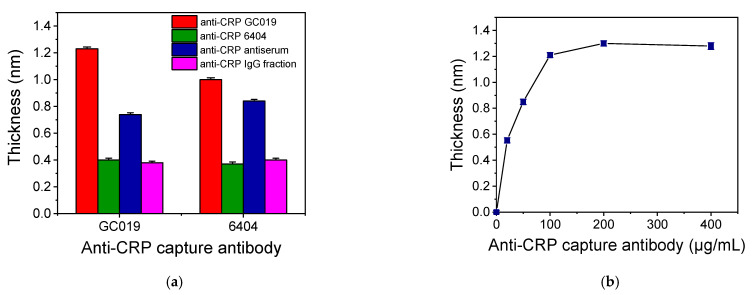
(**a**) Sensor responses obtained for a calibrator containing 100 ng/mL CRP using biochips coated with two different anti-CRP antibodies (GC019 or 6404) at a concentration of 100 μg/mL in combination with four different detection antibodies all at a concentration 10 μg/mL. Each point is the mean value of three measurements ± SD. (**b**) Sensor responses obtained for a calibrator containing 100 ng/mL CRP from chips coated with capture anti-CRP antibody at concentrations ranging from 20 to 400 μg/mL. Each point is the mean value of three measurements ± SD.

The detection of CRP in human plasma using the proposed WLRS device is based on a label-free two-site sandwich immunoassay. As it is schematically depicted in Figure 1d, the assay involved two steps, the first one being the reaction of the CRP molecules in calibrator or sample with the immobilized onto the chip capture antibody and the second one the binding of the detection antibody onto the immunoadsorbed CRP molecules. For the development of the immunoassay, at first, several antibodies were tested as capture and detection antibodies, respectively, in order to select the most appropriate antibody pair. More specifically, a goat polyclonal affinity purified antibody (GC019) and a mouse monoclonal antibody (6404) against CRP were tested both as capture and detection antibodies, while a goat anti-CRP antiserum and a goat anti-CRP IgG fraction were tested only as detection antibody. In all cases, the concentration of the capture and detection antibodies was 100 μg/mL and 10 μg/mL, respectively. The sensor responses obtained for a calibrator containing 100 ng/mL CRP are provided in Figure 2a. As shown, the highest response was obtained when the affinity purified goat polyclonal antibody (GC019) was used both as a capture and detection antibody. It should be noted that all antibody combinations provided zero calibrator responses that could not be distinguished by the baseline fluctuation. Thus, the affinity purified goat polyclonal anti-CPR antibody was selected for assay development and the optimum antibody concentration for coating of the chips was determined. For this purpose, chips coated using anti-CRP antibody solutions with concentration ranging from 20 to 200 μg/mL were tested using a calibrator containing 100 ng/mL CRP, while the detection antibody concentration was in all cases 10 μg/mL. The related WLRS sensor responses are presented in Figure 2b. The response increased as the antibody concentration in the coating solution increased, reaching maximum plateau values at concentrations equal to or higher than 100 μg/mL. Therefore, this concentration was adopted in the final protocol for immobilization of capture antibody. Using the selected capture antibody concentration, the detection antibody concentration was also optimized. It was found that concentrations equal to or higher than 10 μg/mL provided maximum plateau values for reaction duration equal to or higher than 5 min. Thus, a 10 μg/mL concentration was selected for the detection antibody.

**Figure 3:**
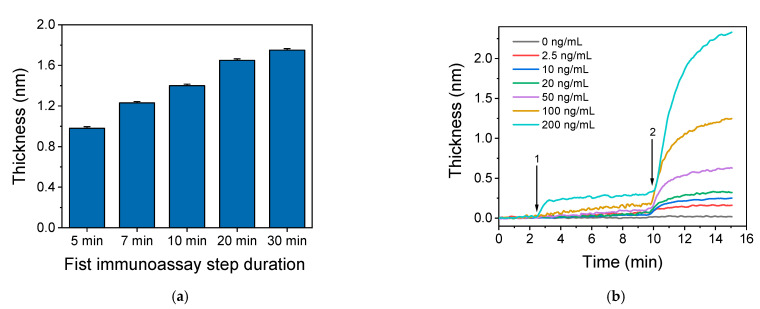
(**a**) Sensor responses obtained upon running a 100 ng/mL CRP calibrator for 5 to 30 min followed by 5-min reaction with a 10 μg/mL anti-CRP detection antibody solution. Each point is the mean value of three measurements ± SD. (**b**) Real-time responses obtained upon running over the chip for 7 min CRP calibrators with concentration ranging from 2.5 to 200 ng/mL (arrow 1 to 2) followed by a 10 μg/mL anti-CRP antibody solution for 5 min (arrow 2 to end of run).

Once the capture and detection antibody concentrations were selected, the duration of each immunoassay step was determined. For this purpose, duration times of 5, 7, 10, 20 and 30 min were investigated for the first assay step keeping the second step duration set at 5 min. As shown in Figure 3a, as the duration of the first immunoreaction step, i.e., the reaction of CRP molecules with the immobilized onto the chip capture antibody, increased, the sensor response increased, reaching plateau values after 20 min of reaction. Nonetheless, the sensor response increased approximately 30% when the duration of the first immunoreaction increased from 7 min to 30 min. In order to investigate if this immunoreaction duration could provide for a wide dynamic range, calibrators with CPR concentration ranging from of 2.5 to 200 ng/mL were run over the biochip for 7 min followed by a 5-min reaction with the detection antibody. The results presented in Figure 3b indicate that in case of high concentration CRP calibrator, i.e., 200 ng/mL, the sensor response obtained during the first step reached the maximum value within approximately 3 min. Regarding the lower CRP concentrations, the response increased continuously and no plateau values were obtained after 7 min of reaction. However, the responses obtained after reaction for 5 min with the detection antibody allowed good discrimination of the calibrators. Thus, a 7 min duration for the first step allowed for a wide assay dynamic range. From the real-time responses presented in Figure 3b, it can be also concluded that, for all CRP calibrators maximum plateau values were obtained for a 5-min duration of the second immunoassay step. In fact, increase of duration of the reaction with the detection antibody from 5 to 10 min resulted in a marginal sensor response increase of about 5%. Therefore, the 5 min duration time of the second assay step was selected for the final protocol, in order to minimize as much as possible, the overall analysis time.

**Figure 4:**
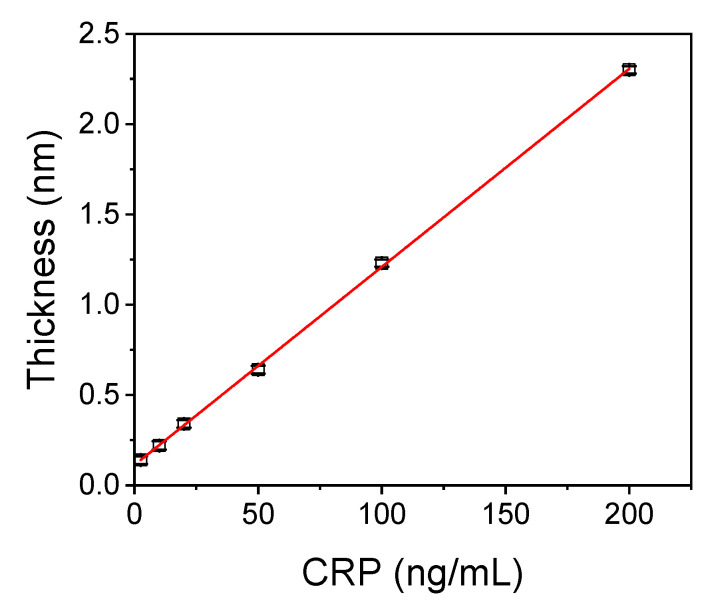
Typical linearized CRP calibration curve obtained with the WLRS bioanalytical system. Each point is the mean value of four replicate measurements ±SD.

The calibration curve obtained using the 12-min immunoassay protocol (15 min including the 3 min baseline equilibration with assay buffer at the start of each run) is presented in Figure 4. The linear regression equation of the calibration curve was: Sensor response = 0.01 (±0.001) concentration + 0.11 (±0.01), and the correlation coefficient was R^2^ = 0.9996. The detection limit (LOD) of the assay was calculated as the concentration corresponding to +3SD of 10 replicate measurements of zero calibrator and was 1.0 ng/mL. Respectively, the limit of quantification (LOQ) was calculated as the concentration corresponding to +6SD of 10 replicate measurements of zero calibrator, and was 2.5 ng/mL.

The linear regression equation was imported in the software of the WLRS device and used to determine the concentration of CRP in human plasma samples taking into account the sample dilution with assay buffer. Thus, prior to determination of CRP in human plasma samples, the effect of sample dilution to sensor response was determined.

### 3.2. Sample Dilution Effect on CRP Assay

**Figure 5:**
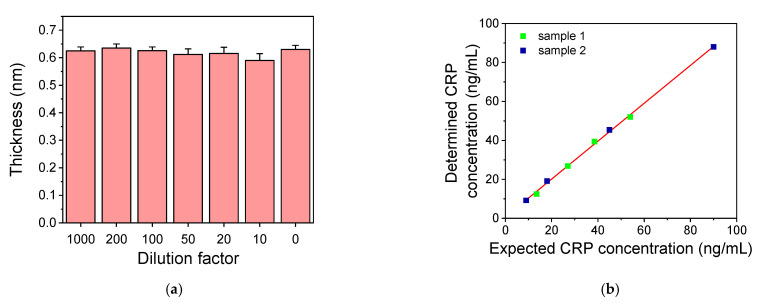
(**a**) Sensor responses obtained upon running a 50 ng/mL CRP solution prepared in assay buffer or in CRP-free human plasma diluted 10–200 folds with assay buffer. Each point is the mean value of three measurements ± SD. (**b**) Plotting of CRP concentration values determined in two plasma samples of known concentration after sequential dilution with assay buffer versus the expected CRP values in the diluted samples.

As discussed in the introduction, CRP is an inflammatory biomarker with values in human blood serum or plasma that can extend over a wide range. In healthy individuals, CRP concentration is usually under 5 μg/mL, while during acute inflammation, it can increase even 1000-fold. On the other hand, in some pathological conditions, like cardiovascular ones, the detection of CRP concentrations at concentration levels lower than 2 μg/mL is desirable for the prognosis of these conditions. In order to cover this extended concentrations range in human serum/plasma samples, dilutions of them is often applied. In the present work, blood plasma was selected as the medium for determination of CPR. This was done in order to be able to extend the application of the developed system to the determination of other markers related to cardiovascular diseases prognosis and/or diagnosis such as d-Dimers, which can be only measured in plasma. The selection was justified by the fact that the CRP values determined in serum and plasma samples from the same individuals employing the highly sensitive CRP Abbott assay were in good agreement. To evaluate the effect of human plasma on the developed CRP assay, human plasma free of CRP was spiked with the appropriate amount of CRP so as after dilution of 10 to 1000 folds with assay buffer the final CRP concentration to be in all cases 50 ng/mL. The sensor responses obtained for these calibrators were compared with the response obtained for the 50 ng/mL CRP solution prepared in assay buffer (Figure 5a). As shown, the responses obtained for all the plasma dilutions were the same with the response provided for the CRP solution prepared in assay buffer. Consequently, since there is no interference from plasma, each of these dilutions can be applied for the CPR concentration determination in human plasma samples, provided that the concentration of the diluted plasma is within the dynamic range of the assay. To verify this, two plasma samples (with known CRP concentration values) were analyzed after different dilutions to evaluate the dilution linearity of the assay. The first sample had CRP concentration of 2.7 μg/mL and was assayed after 50, 70, 100 and 200-fold dilution with assay buffer, while the second sample had CRP concentration of 0.9 μg/mL and was diluted 10, 20, 50 and 100 folds. In Figure 5b, the CRP values determined in the diluted samples where plotted against the values expected in the diluted samples based on their initial value. The linear regression equation of the curve was y = 0.97 (±0.01) x + 0.69 (±0.66) and the correlation coefficient was R^2^ = 0.998, indicating the high dilution linearity of the developed assay. Thus, by applying a 20-fold sample dilution, the assay working range extends from 0.05 to 4.0 μg/mL, covering mostly the range relevant to cardiovascular disease prognosis, while if a 1000-fold dilution is considered, the range extends from 2.5 to 200 μg/mL.

### 3.3. Analytical Evaluation

The reproducibility and the accuracy of the results of the CPR assay run in the WLRS PoC system were evaluated. Reproducibility was determined by repetitive measurements of three plasma samples corresponding to three different CRP concentration levels to cover the assay dynamic range. The intra-assay coefficient of variation (CV) values obtained from four repetitive measurements of each sample within the same day ranged from 5.4 to 7.3%, while the inter-assay CV values obtained from four measurements carried out in four different days in a period of 20 days ranged from 6.8 to 8.1%.

The accuracy of the assay was evaluated through recovery experiments. For this purpose, three plasma samples were analyzed to determine their CRP concentration and then spiked with known concentrations of CRP and analyzed again. The percent recovery (%R) of exogenous added CRP was determined according to equation:

$$\%R=\frac{CRP\;concentration\;after\;addition-CRP\;concentration\;prior\;to\;addition}{CRP\;concentration\;added}$$

As presented in Table 1, the recovery values ranged from 87.0 to 116%, indicating the good accuracy of the CRP assay.

**Figure 6:**
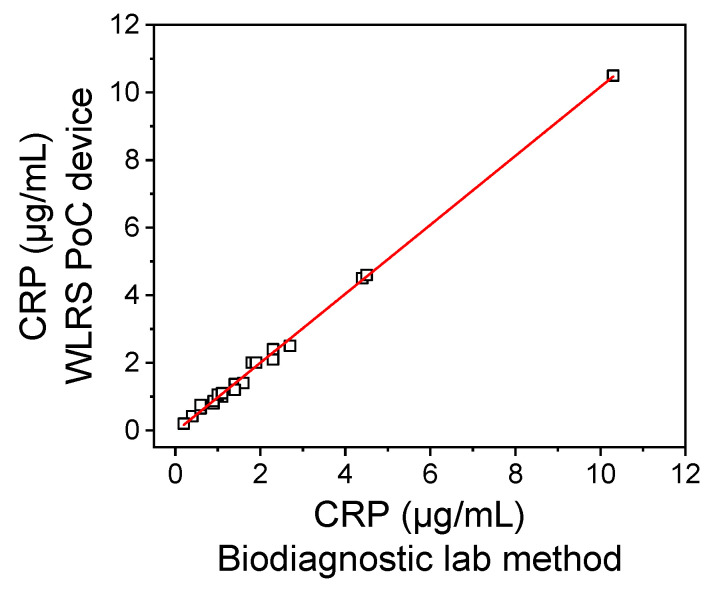
Correlation of CRP concentrations determined in 20 plasma samples from anonymous donors by the assay run on the WLRS PoC device with those obtained for the same samples by the diagnostic laboratory method.

The accuracy of the developed CRP assay was also evaluated by analyzing 20 plasma samples from anonymous donors and comparing the values determined with those provided for the same samples by a diagnostic laboratory method. In Figure 6, the correlation of CRP concentrations determined in the plasma samples by the WLRS device with those measured by the method of the diagnostic laboratory is presented. The equation of the linear regression curve was y = 1.021 (±0.012) x − 0.041 (±0.038) and the correlation coefficient was R^2^ = 0.996, indicating a very good agreement between the values determined with the developed method and those determined with the diagnostic laboratory method. These results demonstrated the accuracy of the measurements performed using the developed WLRS PoC device in analysis of human plasma samples. It should be noticed that the method developed could be applied for determination of CRP concentration in serum samples with the same accuracy.

## 4. Discussion

The WLRS detection principle has been successfully implemented for the immunochemical detection of various substances, both of low and high molecular weight, applying different immunoassay formats [22,28,32,33,34,35]. The main advantages of this technology can be summarized to the following: fast and accurate bioanalytical results, real-time signal monitoring, ease of use, and low cost of consumables and instrumentation. The WLRS instrumentation consists of a visible/near infrared light source, a miniaturized spectrometer, and a reflection probe that delivers the light emitted by the source to the biochip surface and collects the reflected light directing it to the spectrometer.

In the current work, a fully automated PoC system based on the WLRS methodology has been designed for operation at clinical settings were the only intervention by the user is the insertion of the biochip and the loading of the necessary reagents, while all the steps for assay execution are automatically handled by just clicking a button. Thus, in the system developed all optical components, the pump, a carousel for loading the reagents’ solutions required to run the assay and a sampler have been integrated in a compact instrument. The improvements regarding the optical system included the stabilization of light source for long-term operation and the selection of spectral range with respect to spectrometer implemented. In particular, the light source has been equipped with an electronic circuit for stabilized light emission both in terms of spectral content and intensity and for long term operation (>10,000 h). On the other hand, the spectrometer has been tuned in a carefully selected narrow spectral range (450–650 nm) that allowed for the accurate recording of the minute spectral shifts that are caused by the growth of biomolecular layer thickness onto the SiO_2_/Si biochip due to immunoreactions. Thanks to this design, the reference spectrum could be acquired once per month of operation and applied for daily usage. The chip design was not altered; more specifically, the thickness of SiO_2_ layer was kept at ~1000 nm since it was determined by previous studies as the optimum one with respect to sensor response amplitude, but also because standard microelectronic processes easily realize such a thickness. The microfluidic chamber design was also not altered, since the old design secured continuous operation without any leak throughout the assay. The docking station was designed to allow for facile insertion of the biochip on the designated slot through a sliding platform aiming to facilitate the use of the system by non-experts.

The fluidic module was designed taking into account the need for automated operation and easy loading of the vials with the reagents required for the performance of the immunoassay in the dedicated positions. Thus, a carousel that accommodated up to four vials of various volume that could be loaded with different reagent solutions was integrated onto the system along with the sampler and a PC-programmable micro-pump for the supply of these solutions at the desired flow-rate. The programming of the fluidic module by the dedicated software enabled the selection of both the duration and flow rate with which each one of the different immunoassay steps would be performed, endowing the system with high versatility. The dedicated software controlled not only the fluidic module, but it also provided for the reflected spectrum recording and its transformation in real-time to biomolecular layer thickness and finally to CRP concentration in the sample by a single button operation.

In more detail, the operations performed by the software developed included: (a) the normalization of the reflectance spectrum with respect to dark (received with the light source turned-off) and the reference spectrum (received from a plain silicon chip), (b) the fitting of the normalized reflectance spectrum by the appropriate physical model and algorithm for the accurate calculation of thickness of biomolecular layer created in the course of the immunoreactions onto the chip surface, and finally, (c) the calculation of the CRP concentration in the sample analyzed using the linear regression equation determined by correlating the response of the chip to the analyte concentration in the calibrators. The two first operations took place in real-time, and the response was depicted during the assay in the PC screen, while the third operation was performed as soon as the assay sequence was completed.

Using the WLRS PoC system, an assay for the determination of CRP in human plasma samples was developed and its performance was compared with commercially available PoC systems including handheld and bench-top devices. Table 2 contains information about the assay method implemented, along with the type and volume of the sample, the analytical characteristics, i.e., the analysis time and the dynamic range, and the device weight based on literature data as well on data provided by the manufacturers of these devices [12,17,31]. All devices listed require simple sample preparation and short analysis time. Consequently, they can be used for CRP determination at the Point-of-Need, offering shorter turnaround times compared to larger central hospital laboratory systems.

Regarding the proposed bench-top device, the analysis time is comparable to those of the commercial ones, while the method’s LOQ is lower, providing a dynamic range that covers a wide range of concentrations that not only includes normal values, but also patients with acute inflammation incidents, like sepsis, or cardiovascular diseases. This was achieved through the implementation of a two-step assay that provided a LOQ well below the CRP concentration cut-off in healthy individuals, but also absence of high-dose hook effect phenomena, allowing determination of high CRP concentrations just by adjusting the sample dilution. As presented in Table 2, the system developed has been evaluated for plasma and serum samples analysis; however, whole blood samples can also be analyzed, as it has already been presented in our previous report [35]. Regarding the size and the weight of the bioanalytical system, although appropriate for PoC application, they can be further reduced, especially through the minimization of the sampling carrousel system and the implementation of a lighter case. In addition, the automation level and software can be further improved regarding both the raw data management and the user-device interface.

The developed WLRS PoC system can be applied in its current form to perform any immunoassay procedure, either for determination of analytes of clinical interest or analytes related to food safety or environmental samples.

## Figures and Tables

**Table 1:** Percent recovery values of exogenous added CRP to three human plasma samples.

Sample#	Amount Added (μg/mL)	Amount Determined (μg/mL)	% Recovery
1	0	0.53	-
	0.25	0.79	104
	0.50	1.1	114
	1.0	1.4	87.0
2	0	0.92	-
	0.50	1.5	116
	1.0	1.9	98.0
	2.0	2.8	94.0
3	0	1.8	-
	1.0	2.9	110
	2.0	4.7 ^1^	95.0
	5.0	7.7 ^1^	98.0

^1^ A 100-fold dilution was employed for these samples.

**Table 2:** Comparison of the proposed PoC device with commercially available PoC devices for the determination of CRP.

Commercial Device	Method	Sample Type	Sample Volume (μL)	Analysis Time (min)	Dynamic Range (μg/mL)	Device Weight (kg)
PATHFAST (Mitsubisi Chemical)	chemiluminescence enzyme immunoassay	whole blood plasma/serum	100	<17	0.05–30	28
AFIAS (Boditech)	fluorescent solid-phase sandwich immunoassay	whole blood plasma/serum	10	3	0.5–200	15.1
iChroma II (Boditech)	immunochromatographic fluorescent	whole blood plasma/serum	10	3	2.5–300	1.3
AQT90 Flex (Radiometer)	solid-phase sandwich immunoassay	whole blood plasma	2000	<13	5–500	35
SMART (Eurolyser)	latex enhanced immunoturbidimetric assay	whole blood plasma/serum	5	3–4	2–240 0.5–120	3.4
CUBE (Eurolyser)	latex enhanced immunoturbidimetric assay	whole blood plasma/serum	5	3–4	2–240 0.5–120	2.4
Afinion™ (Abbott)	solid-phase immunoassay	whole blood plasma/serum	2.5	3–4	5–200 5–160	3.4
Microsemi LC-667G (Horiba)	immunoturbidimetric assay	whole blood plasma/serum	18	4	0–200 0–150	19
Innovastar^®®^ (Diagnostic Systems GmbH)	immunoturbidimetric assay	whole blood plasma	10	6.5	5–400 2–160	4
Spinit^®®^ (biosurfit SA)	Surface Plasmon Resonance immunoassay	whole blood plasma/serum	8	<4	2–300	4.1
QuickRead go (Aidian)	immunoturbidimetric assay	whole blood plasma/serum	-	2	5–200	1.7
Nano-Check™ (Nano-Ditech)	immunochromatographic assay	whole blood plasma/serum	15	15	0.5–20	-
Proposed device	WLRS solid-phase sandwich immunoassay	plasma/serum	10	12	0.05–200	10

## Data Availability

The data presented in this study are available on request from the corresponding author. The data are not publicly available due to privacy issues.
